# Aging Alters Functionally Human Dermal Papillary Fibroblasts but Not Reticular Fibroblasts: A New View of Skin Morphogenesis and Aging

**DOI:** 10.1371/journal.pone.0004066

**Published:** 2008-12-30

**Authors:** Solène Mine, Nicolas O. Fortunel, Hervé Pageon, Daniel Asselineau

**Affiliations:** L'Oréal, Sciences du Vivant, Clichy, France; Duke University, United States of America

## Abstract

Understanding the contribution of the dermis in skin aging is a key question, since this tissue is particularly important for skin integrity, and because its properties can affect the epidermis. Characteristics of matched pairs of dermal papillary and reticular fibroblasts (Fp and Fr) were investigated throughout aging, comparing morphology, secretion of cytokines, MMPs/TIMPs, growth potential, and interaction with epidermal keratinocytes. We observed that Fp populations were characterized by a higher proportion of small cells with low granularity and a higher growth potential than Fr populations. However, these differences became less marked with increasing age of donors. Aging was also associated with changes in the secretion activity of both Fp and Fr. Using a reconstructed skin model, we evidenced that Fp and Fr cells do not possess equivalent capacities to sustain keratinopoiesis. Comparing Fp and Fr from young donors, we noticed that dermal equivalents containing Fp were more potent to promote epidermal morphogenesis than those containing Fr. These data emphasize the complexity of dermal fibroblast biology and document the specific functional properties of Fp and Fr. Our results suggest a new model of skin aging in which marked alterations of Fp may affect the histological characteristics of skin.

## Introduction

Chronological aging is described as a time-dependant biological process leading to gradual changes in the structure and functions of all tissues that compose an organism. These modifications tend to decrease the capacity for adaptive responsiveness and wound healing, and therefore enhance its susceptibility to disorders and death. The genetic program that determines life span and aging is investigated using models of different species, including yeasts (Saccharomyces cerevisiae), worms (Caenorhabditis elegans), insects (Drosophila melanogaster), and mammals (Mus musculus) [1]. In humans, genetic diseases which display clinical signs of premature aging such as progeroid syndromes are an important source of information to identify molecular effectors involved in aging [2]. The importance of specific allelic polymorphisms in the predisposition of human beings to longevity or resistance to age-related disorders has been emphasized by studies of centenarians [3]. In addition to intrinsic genetic factors, chronological aging is controlled through epigenetic mechanisms and is affected by environmental parameters including nutrition [4], which clearly leads to consider this biological process as a complex multifactorial phenomenon.

Skin represents a valuable model to study aging in humans, since it is widely affected by this process and is easily accessible. Modifications related to aging are particularly visible in human skin, which becomes wrinkled, lax, dry, and irregularly pigmented over time [5]. Aged skin is characterized by a flattening of the dermal-epidermal junction, a marked atrophy and a loss of elasticity of the dermal connective tissue [6], associated with a reduction and disorganization of its major extracellular matrix components, such as collagen and other elastic fibers [7], proteoglycans and glycosaminoglycans [8]. A histological characteristic of chronological aging in the epidermis is a decrease of tissue thickness [7]. Solar radiations are particularly studied as environmental factors promoting skin aging [9] and carcinogenesis [10]. UV lights have been shown to affect both epidermal keratinocytes [11] and dermal fibroblasts [12]. However, at present, age-related modifications of the different dermal fibroblast populations and the consequent effects on skin aging remain poorly understood.

Human fibroblasts can be isolated from various connective tissues, including skin [13]. These cells exert important functions in the regulation of tissue structure and cellular microenvironment, by the production of extracellular matrix proteins [14], cytokines, growth factors [15], and matrix metalloproteinases [16]. The pioneering work of Hayflick has revealed that serially cultivated fibroblasts exhibit a limited capacity to divide *in vitro* [17], [18]. This experimental model, termed replicative senescence, has been extensively investigated to approach *in vitro* the biology of fibroblast aging and senescence, at the cellular and molecular levels. Alterations of fibroblast properties, associated with aging or senescence, have been studied either in long-term cell cultures [19]–[21], or by using fibroblasts from skin biopsies derived from donors with increasing age [22]. The observed age-related changes in fibroblasts include cell morphology, metabolism [19], reduced proliferative potential [19], [22], [23], loss of responsiveness to growth factors [24], decline in the production of extracellular matrix proteins such as type I and III collagens [25], and overexpression of proteases involved in the degradation of the extracellular matrix [26]. Reduction of telomere length [27], accumulation of free radical-induced DNA damages, and decrease of DNA repair [28], are molecular mechanisms associated with and promoting senescence. The above described modifications of fibroblast properties observed throughout serial *in vitro* expansion may all more or less participate to the *in vivo* age-related changes of the skin.

A wide diversity of characteristics have been reported for fibroblasts derived from different tissues [29]. Even in a single tissue, heterogeneity is an important intrinsic property of fibroblasts residing at different micro anatomical localizations, which has to be taken into consideration when studying cellular aging. In human skin, it has been reported that cultured fibroblasts isolated from the upper and the deeper dermis, at the same anatomic site, exhibit distinct properties in regard to cellular morphology and proliferative potential [30], [31], elaboration of the extracellular matrix [32]–[34], the production [35] and response [36], [37] to growth factors and cytokines. However, little is currently known concerning the specific functions of these different fibroblast subpopulations, and their respective modifications and fate during the process of skin aging.

The aim of the present work was to investigate the age-related modifications of distinct human dermal fibroblast populations and their possible implication in skin aging. Fibroblasts were obtained from the superficial layer of the dermis, named “papillary dermis”, and from the deeper dermal layer, named “reticular dermis”. Matched pairs of papillary (Fp) and reticular fibroblasts (Fr), derived from donors of different ages were studied and analyzed for cellular morphology, secretion of growth factors and cytokines, production of matrix proteases, growth characteristics, and their capacity to promote epidermal morphogenesis.

## Materials and Methods

### Tissue and cell sample preparation

Samples of human mammary skin (sun-protected skin areas) were obtained from adult Caucasian women, after plastic surgery. Informed consent was obtained before tissue collection, according to European guidelines. Age of donors ranged from 19 to 74 years. After removing the subcutaneous tissue, skin samples were sliced according to depth using a dermatome. The upper part of skin samples (from the surface to a 0.3 mm depth) contained the epidermis and the papillary dermis. The deeper part of skin samples (depth >0.7 mm) corresponded to the reticular dermis. Quality of full skin samples and sections was monitored by histological analysis. Collagen fibers were stained with Sirius red (Interchim, Montluçon, France). The epidermis was separated from papillary dermis by dissection, after treatment with 0.25% trypsin for 1.5 hours at 37°C (Invitrogen, Carlsbad, CA).

For the preparation of papillary (Fp) and reticular fibroblasts (Fr), pieces of deep and superficial dermis were placed in culture in MEM supplemented with 10% fetal bovine serum (FBS) (D-Dutscher, Brumath, France), penicillin-streptomycin (20 U/ml) (Biochrom, Cambridge Ldt, UK), and glutamine (2 mM) (Invitrogen, Carlsbad, CA), and maintained at 37°C in a 90% humidified atmosphere containing 5% CO_2_. After migration of fibroblasts outward dermal explants, these cells were amplified in culture and stored in liquid nitrogen as frozen aliquots until use. Match pairs of Fp and Fr were used for the different investigations at the same early passage.

The keratinocytes used in this study were from the epidermis of a single skin sample from a 28 years old donor. Epidermal pieces were dissociated by incubation in 0.25% trypsin for 1.5 hours at 37°C (Invitrogen, Carlsbad, CA). Keratinocytes were plated on a feeder-layer of growth-arrested 3T3 murine fibroblasts, and amplified according to the method described by Rheinwald and Green [38]. Keratinocytes were then frozen in liquid nitrogen until use for three dimensional skin reconstruction experiments.

### Quantification of secreted cytokines, MMPs and TIMPs

Samples of Fp and Fr were plated at a density of 1×10^4^ cells/cm^2^ in duplicate 60 mm culture dishes in a medium composed of MEM supplemented with 10% fetal bovine serum (FBS) (D-Dutscher, Brumath, France), penicillin-streptomycin (20 U/ml) (Biochrom, Cambridge Ldt, UK), and glutamine (2 mM) (Invitrogen, Carlsbad, CA). Culture medium was renewed every 3 days after fibroblast plating. To minimize the quantities of endogenous cytokines, growth factors, matrix metalloproteinases (MMPs) and tissue inhibitor of metalloproteinases (TIMPs) present in the medium, cultures were maintained in the presence of a low serum concentration (1%) the 3 last days before the analysis of secretion profiles of Fp and Fr (from day 6 to day 9). At day 9, supernatants from Fp and Fr cultures were sampled and processed for the quantification of cytokines, MMPs, and TIMPs by ELISA: interleukin-6 (IL-6), monocyte chemoattractant protein-1 (MCP-1), vascular endothelial growth factor (VEGF), keratinocyte growth factor (KGF), MMP-2, MMP-3, TIMP-1 and TIMP-2 (Human Quantikine kits, R&D Systems, Minneapolis, MN), MMP-1 (Biotrack kit, Amersham Biosciences, Buckinghamshire, UK). Background concentrations of cytokines, MMPs and TIMPs present in the culture medium containing 1% FCS in the absence of cells were found very low, often below the detection threshold of quantification assays. When detectable, these values were deduced from concentrations measured in cell supernatants. Values were then normalized according to cell numbers counted in the respective cultures and expressed in pg/ml (cytokines) and ng/ml (MMPs and TIMPs) per 1×10^5^ cells.

### Flow cytometry analysis

Morphological characteristics of young and old Fp and Fr were analyzed by flow cytometry (FACS Aria, BD Biosciences, San Jose, CA). Cultures of Fp and Fr from 3 matched pairs of young and 3 of old donors were trypsinized 7 days after plating, and re-suspended in PBS supplemented with 0.2% bovine serum albumin (BSA) at 4×10^5^ cells/ml. The respective cell samples were analyzed for size (forward-scatter, FSC) and granularity (side-scatter, SSC). Results were referred to viable cells, discriminated from dead cells by 7 AAD staining (eBioscience, San Diego, CA).

### Short term growth

Previously amplified fibroblasts in primary culture were plated in triplicates into 10 cm^2^ wells at a density of 1×10^4^ cells/cm^2^, and cultured in a medium composed of MEM supplemented with 10% FBS (PAN Biotech GmbH, Aidenbach, Germany), penicillin-streptomycin (20 U/ml) (Biochrom, Cambridge Ldt, UK), and glutamine (2 mM) (Invitrogen, Carlsbad, CA). Cell counts were performed at days 7, 14, and 21 after culture initiation. Cellular expansion values corresponded to numbers of cells counted after 7, 14 and 21 days of culture divided by the number of cells initially plated.

### Colony assays

Fibroblasts from the different individual samples were plated at the low density of 100 cells per 100 mm diameter culture dish (Corning, New York, NY). Cultures were performed for 12 days in a medium composed of MEM supplemented with 10% fetal bovine serum (FBS) (Sigma, St Louis, MO), penicillin streptomycin (20 U/ml) (Biochrom, Cambridge Ldt, UK), and glutamine (2 mM) (Invitrogen, Carlsbad, CA), and maintained at 37°C in a 90% humidified atmosphere containing 5% CO_2_. Medium was completely renewed every 4 days. Cultures were fixed in ethanol (70%) and stained with Eosin (RAL, Bordeaux, France) and Methylen Blue (RAL, Bordeaux, France). Colonies were counted and photographed under an inverted microscope coupled with a CCD camera (DMIL, Leica Microsystems, Wetzlar, Germany). The area occupied by cells in individual colonies was scored using Image J software (NIH, Bethesda, MA). Colony counts were based on the analysis of triplicate dishes. Colonies were classified according to the surface occupied by cells and separated into 3 groups: large colonies (>1.5 mm^2^), colonies of intermediate size (0.5–1.5 mm^2^), and small colonies (<0.5 mm^2^). Correspondence between the surface of colonies and cell numbers was determined by cell counting on micro-photographs of fixed colonies.

### Three-dimensional skin reconstruction and xenograft experiments

Reconstructed skins were prepared as previously described [39]. Briefly, fibroblasts (1×10^6^ cells per sample of reconstructed dermis) were embedded into a bovine type I collagen gel (Symatèse, Chaponost, France). Diameters of each dermal equivalent (lattice) were measured using a millimeter scale during the 4 days of lattice contraction. Thereafter, keratinocytes (5×10^4^ cells per sample of reconstructed epidermis) were seeded onto the lattices, stuck to the bottom of 60 mm diameter Petri dishes. Cultures were maintained for 1 week immersed in a medium composed of MEM (Invitrogen, Carlsbad, CA) supplemented with 10% FBS (Sigma, St Louis, MO), Epidermal Growth Factor (EGF) (10 ng/ml) (BD Biosciences, San Jose, CA), Hydrocortisone (0.4 µg/ml) (Sigma, St Louis, MO), and Cholera Toxin (0.1 nM) (Biomol Int., Plymouth, PA). Complete epidermal stratification and full differentiation was obtained 1 week after raising the system at the air-liquid interface. During the whole process of skin reconstruction, cultures were maintained at 37°C, in a fully humidified atmosphere containing 5% CO_2_.

Transplantation of human reconstructed skin samples onto immunodeficient athymic mice were performed using a previously published procedure [40]. Experiments were conducted in agreement with the European guidelines (European instruction 86/609), and were approved by an animal care and use committee. Recipient mice were 6-week-old ICO Swiss nu/nu mice. Bandages were maintained at least 1 week after grafting. Human reconstructed skin samples were harvested 5 weeks after grafting, and processed for histological analysis.

### Histology

Samples of human and reconstructed skin were fixed in paraformaldehyde, dehydrated, and embedded in paraffin. Histological examinations of tissue sections were performed after hematoxylin-eosin-saffron (HES) staining, using a Leica DMR light microscope (Leica Microsystems, Wetzlar, Germany). Thicknesses of spinous, granular and cornified layers, as well as total nucleated cell layers, were measured by image analysis using the image J software (16 different quantifications were performed for each experimental condition).

### Immunofluorescence

Samples of reconstructed skin were embedded in Tissue-Tek (Miles Inc., Elkart, IN) and frozen in liquid nitrogen, and cut into 5 µm thickness sections (cryostat, CM3050 S, Leica, Microsystems, Wetzlar, Germany). Filaggrin was stained using a mouse monoclonal antibody (clone BT-576, Biomedical technologies Inc., Stoughton, MA), revealed using a FITC-conjugated rabbit anti-mouse polyclonal antibody (DAKO DK-2600, Glostrup, Denmark). Nuclei were stained using propidium iodide (Sigma, St Louis, MO). Stained tissue section were observed and imaged under fluorescence microscope (DMR, Leica, Microsystems, Wetzlar, Germany).

Cell samples were plated on glass slides in micro-culture chambers (Lab-Tek, Nalgen Nunc international, NY), and cultured for 3 days in MEM / 10% FBS. Microphotographs of cultured cells were taken under light microscopy (DMIL, Leica Microsystems, Wetzlar, Germany). For immunofluorescence labeling, cells were fixed in 4% paraformaldehyde (Carlo erba, Rodano, Ilaly), washed in TBS, and permeabilized in TBS-Tween 0.5% (Sigma, St Louis, MO). Vinculin was stained using a mouse FITC-conjugated monoclonal antibody (Clone hVIN-1, Sigma, St Louis, MO). Actin filaments were stained using TRITC-conjugated phalloidin (Invitrogen, Calsbad, CA). Stained cultured cells were observed and imaged under fluorescence microscope (DMIRB, Leica, Microsystems, Wetzlar, Germany).

### Statistics

Correlation between concentrations of secreted cytokines, MMPs, TIMPs and donor age were estimated by linear regression. Correlation coefficient (R) and statistical significance (P) were considered acceptable when values were R>0.5 and P<0.05, respectively. For the other studies, differences between experimental groups were analyzed using the ANOVA mixed model. Differences were considered as statistically significant when P values were <0.05. When necessary, data were transformed in logarithm to satisfy conditions of normality and homogeneity. When variances were unequal, ANOVA was run on rank-transformed data.

## Results

### Experimental definition of Fp and Fr cells

**Figure 1:**
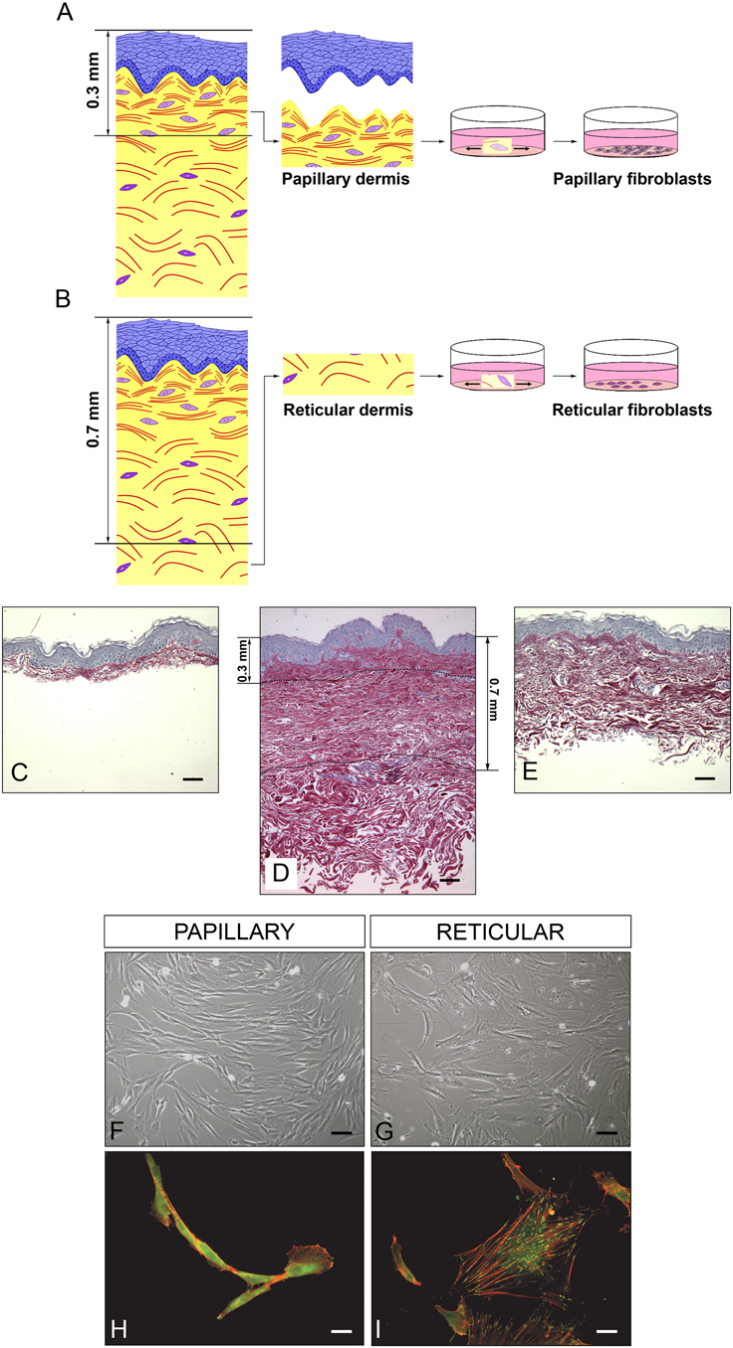
Experimental definition of Fp and Fr cells. (A) and (B), principle of papillary (Fp) and reticular (Fr) fibroblast isolation. (C) Histological preparation of a mammary skin sample after dermatome cutting at a depth of 0.3 mm to obtain the superficial dermis with the epidermis. (D) Full thickness histology of the same skin sample. (E) Histological preparation of the skin sample after dermatome cutting at a depth of 0.7 mm to reach the deep dermis. Scale bars = 50 µm. (F) and (G) Photographs of typical cultures of Fp and Fr in light microscopy. Scale bars = 10 µm. (H) and (I) Fluorescence photographs of Fp and Fr after actin and vinculin immuno-staining. Scale bars = 2.5 µm.

The process used to generate Fp and Fr samples is schematized in Fig. 1A and 1B. Fp and Fr were respectively isolated from superficial dermis (between the surface to 0.3 mm depth) and deep dermis (more than 0.7 mm depth). Cutting at a depth of 0.3 mm allowed the recovery of superficial papillary dermis with the epidermis (Fig. 1C). The skin part that was discarded after cutting at 0.7 mm depth to select the deep reticular dermis is visualized in Fig. 1E. After few days of culture, Fp were characterized by a spindle-shaped morphology (Fig. 1F), although cultured Fr had a stellate morphology (Fig. 1G). Staining of actin indicated a diffuse organization of cytoskeleton in Fp (Fig. 1H). On the contrary, actin filaments were marked in Fr (Fig. 1I). Focal adhesion points, revealed by vinculin staining, were more abundant in Fr (Fig. 1I) than in Fp (Fig. 1H).

### Main histological characteristics of human young and old mammary skin

**Figure 2:**
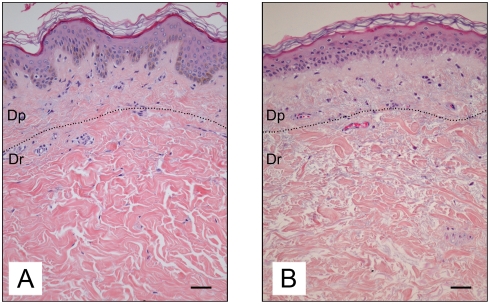
Histological characteristics of young and old human skin. Photographs shown correspond to histological sections of mammary skin biopsies obtained from a 19 year old (A) and a 74 year old donor (B). Sections were stained with hematoxylin, eosin, and saffron (HES). Scale bars = 50 µm. Dp = papillary dermis. Dr = reticular dermis.

The age-related characteristics of human skin are illustrated by the representative histological sections of mammary skin biopsies obtained from a young (19 yr) and an old (74 yr) adult donor (Fig. 2A and 2B, respectively). In general, the epidermis was thicker in young compared to old skin. The rete-ridge structures were clearly observed in sections of young skin, whereas old skin is characterized by a marked flattening of the dermo epidermal junction. A structural disorganization of the collagen fibers was observed in the aged dermis.

### Secretion of cytokines and MMPs/TIMPs by Fp and Fr as a function of donor's age

**Figure 3:**
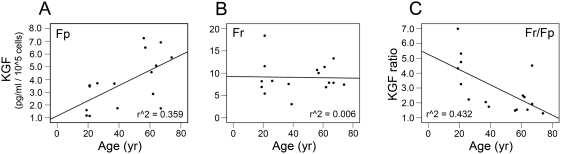
Secretion of KGF by Fp and Fr as a function of donor's age. Evolution of secretion was estimated by linear regression in a cohort of 16 different pairs of Fp and Fr obtained from independent donors, ranging from 19 to 74 years old. Secretion of KGF was quantified. Values are expressed in pg/ml per 10^5^ cells. (A) KGF secretion by Fp samples. (B) KGF secretion by Fr samples. (C) KGF secretion ratio [Fr/Fp].

Age-related secretion of cytokines and MMPs/TIMPs by Fp and Fr was analyzed in a cohort of skin samples from 16 independent donors, ranging from 19 to 74 years old, by linear regression. Age-related evolution of cytokine secretion by Fp and Fr was characterized by distinct profiles. Secretion of KGF increased with age in Fp (Fig. 3A), and remained constant in Fr (Fig. 3B). Consequently, the ratio of KGF secretion by Fr and Fp (Fr/Fp) in paired samples from the same skin biopsy decreased with donor's age (Fig. 3C). The evolution of VEGF secretion resembled that of KGF, however, its up-regulation in Fp from old donors was not statistically significant. MCP-1 secretion was did not vary in Fp, but was decreased with age in Fr. Secretion of IL-6 was not affected by aging in both fibroblast populations. For all analyzed MMPs and TIMPs a progressive increase of secretion level was observed in Fp, whereas no significant alterations with age were detected for Fr. All values are detailed in Table 1.

**Table 1:** Secretion profiles of cytokines, MMPs and TIMPs by Fp and Fr as a function of donor's age.

Category	Specific proteins	Fibroblasts and ratio	R	p-value	Regulation
**Cytokines**	**KGF**	Fp	*0.599	*0.014	*Up-regulation*
		Fr	0.075	0.782	*No regulation*
		Fr/Fp	*0.657	*0.006	
	**VEGF**	Fp	0.38907	0.1364	*Up-regulation*
		Fr	0.105	0.698	*No regulation*
		Fr/Fp	0.41096	0.1138	
	**IL-6**	Fp	0.162	0.55	*No regulation*
		Fr	0.04	0.883	*No regulation*
		Fr/Fp	0.114	0.674	
	**MCP-1**	Fp	0.176	0.515	*No regulation*
		Fr	*0.514	*0.042	*Down-reguation*
		Fr/Fp	*0.615	*0.011	
**MMPs**	**MMP-1**	Fp	*0.531	*0.034	*Up-regulation*
		Fr	0.258	0.335	*No regulation*
		Fr/Fp	*0.690	*0.003	
	**MMP-2**	Fp	*0.533	*0.034	*Up-regulation*
		Fr	0.158	0.559	*No regulation*
		Fr/Fp	0.479	0.0601	
	**MMP-3**	Fp	*0.527	*0.036	*Up-regulation*
		Fr	0.095	0.726	*No regulation*
		Fr/Fp	*0.686	*0.003	
**TIMPs**	**TIMP-1**	Fp	*0.622	*0.010	*Up-regulation*
		Fr	0.096	0.724	*No regulation*
		Fr/Fp	*0.609	*0.012	
	**TIMP-2**	Fp	*0.536	*0.032	*Up-regulation*
		Fr	0.098	0.719	*No regulation*
		Fr/Fp	0.451	0.079	

Evolution with age of the secretion profiles of Fp and Fr was analyzed by linear regression on a cohort of 16 different pairs of fibroblast samples obtained from independent donors, ranging from 19 to 74 years old. Secretion level of specific proteins was considered as significantly modulated with age when the correlation coefficient (R) was found >0.5 (in absolute value) and p<0.05 (*).

### Evolution with age of Fp and Fr morphology

**Figure 4:**
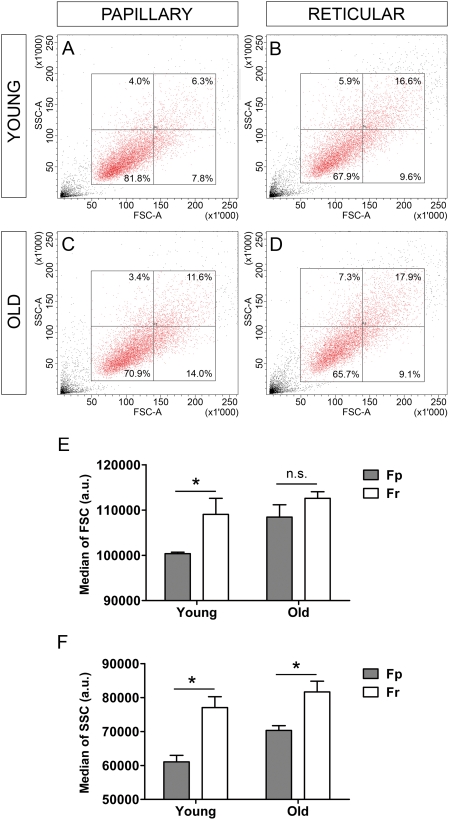
Evolution with age of Fp and Fr morphology. Fp and Fr samples from 3 young donors (19 yr, 21 yr, and 26 yr) and 3 old donors (57 yr, 62 yr, and 74 yr) were examined by flow cytometry according to the morphological parameters of size (forward scatter, FSC) and granularity (side scatter, SSC). FSC versus SSC dot plots corresponding to a typical pair of young [19 yr] Fp (A) and Fr (B), and a typical pair of old [74 yr] Fp (C) and Fr (D). Median FSC and SSC values were determined for the different samples. (E) Comparison of Fp and Fr FSC values in young and old donors (mean±SEM, n = 3). (F) Comparison of Fp and Fr SSC values in young and old donors (mean±SEM) (*p<0.05; n.s. = not significant). a.u. = arbitrary unit.

Populations of Fp and Fr from young (19 yr, 21 yr, and 26 yr) and older donors (57 yr, 62 yr, and 74 yr) were examined by flow cytometry, according to the morphological parameters of size (forward scatter) [FSC] and granularity (side scatter) [SSC]. Dot plots corresponding to Fp and Fr from a typical young donor (19 yr) (Fig. 4A and 4B, respectively), and from a typical older donor (74 yr) (Fig. 4C and 4D, respectively), indicated that all cell samples contained cells with heterogeneous morphologies. However, in the young age group, median FSC (Fig. 4E) and SSC (Fig. 4F) values were significantly lower in Fp than in Fr (p<0.05), indicating that Fp samples contained a higher proportion of small sized cells with low granularity than Fr samples. Morphological differences between Fp and Fr appeared less marked in the older group. Indeed, median SSC values were still significantly lower in old Fp than in old Fr (p<0.05), but this difference was less important. Median FSC values of Fp and Fr were still not significant in the old group.

### Age-related growth characteristics of fibroblasts isolated from papillary (Fp) and reticular (Fr) dermis

**Figure 5:**
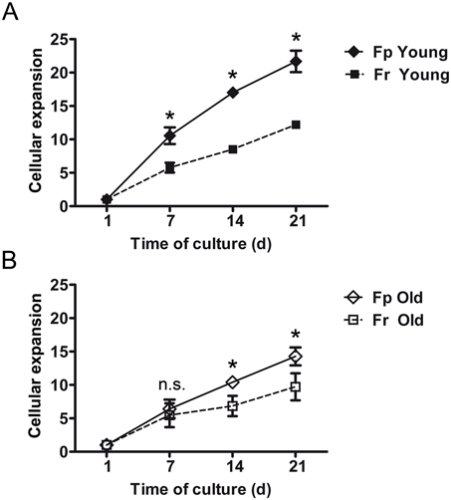
Age-related growth characteristics of Fp and Fr. Fp and Fr samples from 3 young (19 yr, 19 yr, and 26 yr) and 3 old donors (57 yr, 58 yr, and 67 yr) were cultured for 21 days. Cellular growth rate was estimated every 7 days after culture initiation. Data are expressed as cumulative expansion curves (means±SEM, n = 3 independent samples). (A) Comparison of growth rates of Fp and Fr from young donors. (B) Comparison of growth rates of Fp and Fr from old donors (*p<0.05; n.s. = not significant).

Papillary and reticular fibroblasts (Fp and Fr) isolated from skin samples from 3 young (19 yr, 19 yr, and 26 yr) and 3 old individuals (57 yr, 58 yr, and 67 yr), were studied for their respective growth rate in short-term culture. Mean cumulative expansion curves indicated that, in the young age group, Fp exhibited a higher growth rate than Fr (Fig. 5A), in particular when comparing the respective rates at day 7, day 14, and day 21 (p<0.05). In the old age group, this difference was less marked, but still significant at day 14 and day 21 (p<0.05) (Fig. 5B). It is interesting to note that Fr growth rates were not significantly affected by age with a cellular growth index at day 21, for young Fr and old Fr of 12.2±0.5 and 9.07±2.03, respectively. On contrary, a marked decrease of cellular growth index with age was observed in Fp at day 21, with 21.6±1.6 for young and 14.3±1.3 for aged cells (p<0.05).

### Evolution with age of the clonogenic capacity of Fp and Fr

**Figure 6:**
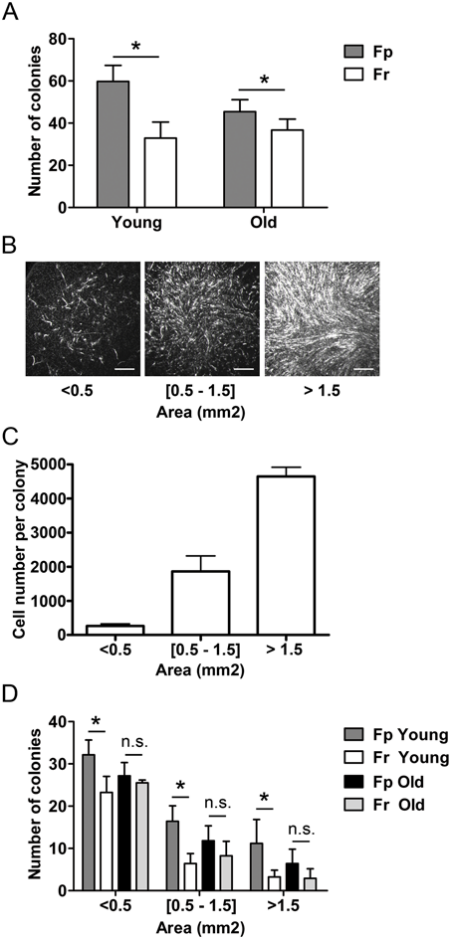
Evolution with age of Fp and Fr clonogenic capacity. The growth capacity of Fp and Fr samples from 4 young (19 yr, 21 yr, 23 yr, and 26 yr) and 4 old donors (57 yr, 60 yr, 63 yr, and 74 yr) was analyzed in term of colony-forming efficiency (CFE) and size of the colonies generated by these different fibroblast samples. (A) Comparison of the CFE of Fp and Fr from young and old donors. Data are expressed as number of colonies obtained from 100 plated fibroblasts (means±SEM, n = 4, independent samples). Colonies were classified according to their individual area (mm^2^). (B) Photographs of colonies of typical ‘small’, ‘intermediate’, and ‘large’ size. Scale bars = 50 µm. (C) Correspondence between colony area (mm^2^) and fibroblast number (means±SEM, n = 10 colonies per size category). (D) Comparison of the sizes of colonies generated by Fp and Fr from young and old donors. The histogram shown corresponds to the number of colonies obtained from 100 plated fibroblasts (means±SEM, n = 4, independent samples), (*p<0.05; n.s. = not significant).

Investigation of the clonogenic capacity of Fp and Fr with aging on cell samples prepared from skin biopsies of 4 young (19 yr, 21 yr, 23 yr, and 26 yr) and 4 old (57 yr, 60 yr, 63 yr, and 74 yr) donors indicated that the colony-forming efficiency (CFE) of Fp was significantly higher than that of Fr, whatever the age of donors (Fig. 6A). This difference was particularly marked in samples from young donors: for an equivalent of 100 plated cells, 59.75±7.6 and 32.97±7.6 colonies were obtained from young Fp and Fr, respectively (p<0.05). The CFE of Fp remained significantly more elevated than that of Fr in old donors, but this difference was reduced: respectively 45.4±5.6 and 36.67±5.2 colonies were obtained / 100 plated Fp and Fr (p<0.05). Colonies produced by the different Fp and Fr samples were then classified according their size (i.e. area occupied by fibroblasts in individual colonies). As shown in Fig. 6B, small colonies (area <0.5 mm^2^) were characterized by a low fibroblast density, whereas cell density was markedly higher in large size colonies (area >1.5 mm^2^). Cell density was intermediate in medium size colonies. Correspondence between colony area (mm^2^) and fibroblast number indicated that small size, medium size, and large size colonies contained respectively 2.66×10^2^±0.61×10^2^, 1.86×10^3^±0.45×10^3^, and 4.65×10^3^±0.27×10^3^ fibroblasts (Fig. 6C). Analysis of colony size also indicated marked functional differences between Fp and Fr in the young age group (Fig. 6D). In particular, the higher clonogenic growth capacity of young Fp, as compared to that of Fr of similar age, was clearly illustrated by the increased number of large size colonies generated by Fp: respectively 11±6 and 3±2 large colonies were obtained / 100 plated young Fp and Fr (p<0.05). This difference in the growth capacity of Fp and Fr was lost with increasing age.

### Age-dependent capacity of Fp and Fr to contract a collagen gel

**Figure 7:**
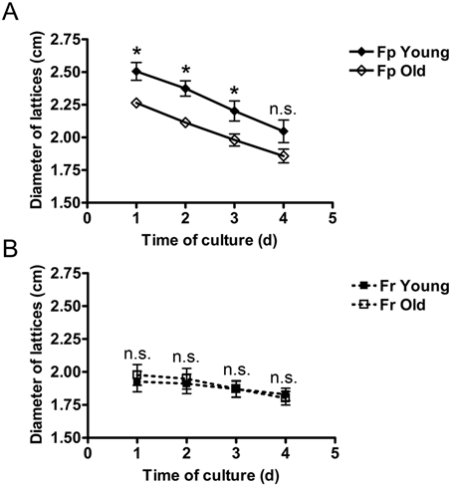
Age-dependent capacity of Fp and Fr to contract a collagen gel. Dermal equivalents were generated with Fp and Fr samples from 3 young (19 yr, 21 yr, and 26 yr) and 3 old donors (57 yr, 62 yr, and 74 yr). Diameter of lattices (cm) was measured at days 1, 2, 3, and 4. (A) Evolution of the diameter of lattices generated with Fp from young and old donors (means±SEM, n = 3, independent samples). (B) Evolution of the diameter of lattices generated with Fr from young and old donors (means±SEM, n = 3). (*p<0.05; n.s. = not significant).

To investigate differences in the capacity of Fp and Fr from young (19 yr, 21 yr, and 26 yr) and old (57 yr, 62 yr, and 74 yr) donors to contract a collagen gel, cells were embedded into type I collagen, and the diameter of dermal equivalents (lattices) was measured from day 1 to day 4 after culture initiation. In the young age group, the diameters of the lattices containing Fr cells were significantly lower than those containing Fp cells at each time point of the study (p<0.05), indicating a higher rate of lattice contraction of Fr. Differences were particularly important at day 1: diameter of lattices containing Fp and Fr were 2.506 cm±0.068 and 1.928 cm±0.078, respectively. Differences in the lattice retraction rate between Fp and Fr became less important with increasing age. At day 1, diameter of lattices containing Fp and Fr were respectively 2.264 cm±0.035 and 1.976 cm±0.078 (p<0.05). Differences were still significant at days 2 and 3, but not at day 4. In general, the lattice contraction capacity of Fr did not change with age (Fig. 7B), whereas in Fp the contraction rate clearly increased with age (p<0.05) (Fig. 7A).

### Age-related impact of Fp and Fr on the epidermal compartment in reconstructed skin

**Figure 8:**
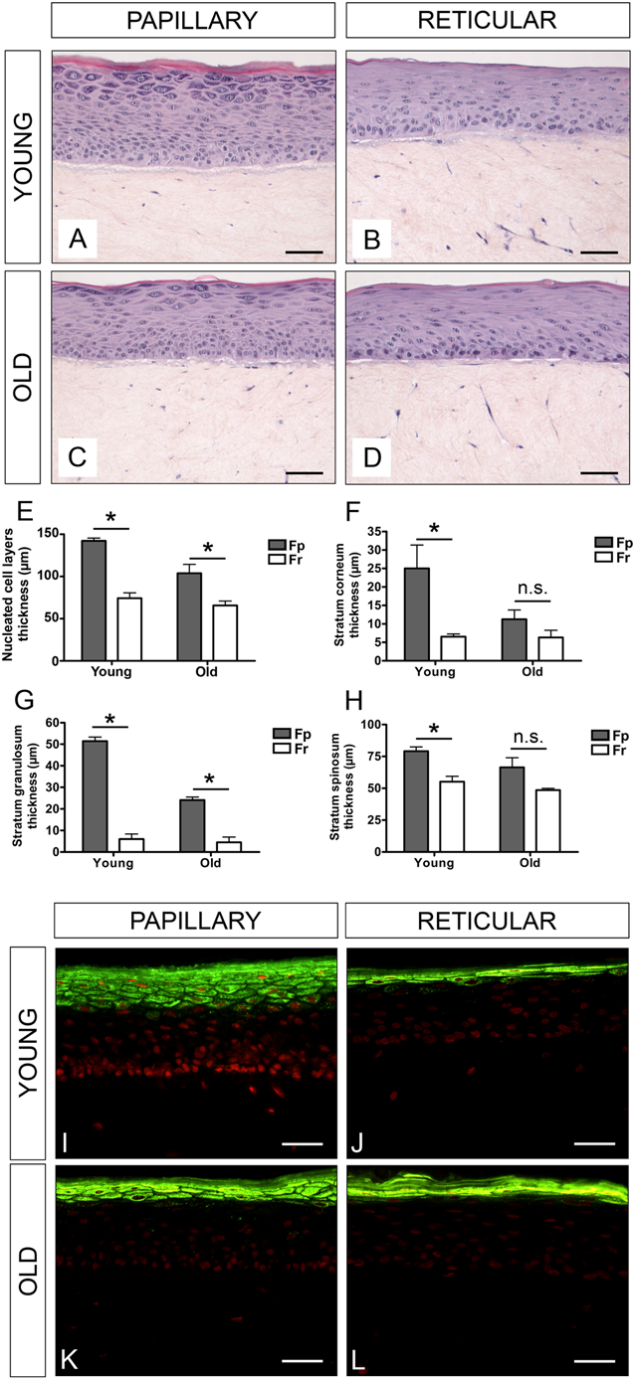
Age-related impact of Fp and Fr on the epidermal compartment in three-dimensional reconstructed skin. Keratinocytes from the same batch were seeded onto dermal equivalents containing Fp or Fr from young or old donors. Typical histological sections of reconstructed skin samples made with collagen contracted lattices containing: (A) young Fp; (B) young Fr; (C) old Fp; (D) old Fr. Measurements of the thicknesses of total nucleated (E), cornified (F), granular (G), and spinous (H) keratinocyte layers in reconstructed skins generated in the presence of Fp or Fr from young or old donors. (I) to (L) Typical expression pattern of filaggrin in reconstructed skin epidermis generated in the presence of Fp or Fr from young or old donors. Scale bars = 50 µm.

The effect of Fp and Fr from young and old donors on skin reconstruction was evaluated in a reconstructed skin model comprising a dermal and epidermal equivalent. Keratinocytes from the same batch were seeded onto dermal equivalents containing either Fp or Fr from young or old donors. Histological sections revealed that young Fp were more potent to promote epidermal morphogenesis than Fr of similar age. Epidermis reconstructed on lattices containing young Fp had more cell layers and was fully differentiated (Fig. 8A), whereas lattices with young Fr generated a thin and incompletely differentiated epidermis (see granular and horny layers) (Fig. 8B). The differentiation of epidermis generated in the presence of old Fp (Fig. 8C) and Fr (Fig. 8D) was equivalently poor, indicating alterations of Fp with age. In particular, epidermis obtained in the presence of young Fp was characterized by higher thicknesses of total nucleated keratinocyte (Fig. 8E), cornified (Fig. 8F), granular (Fig. 8G), and spinous (Fig. 8H) layers, as compared with epidermis generated in the presence of Fr of similar young ages (p<0.05). Differences were less marked when epidermis was generated in the presence of Fp and Fr from older donors. Detection of filaggrin, a marker of differentiated keratinocytes, confirmed the highest capacity of Fp from young donors to promote epidermal morphogenesis and full differentiation, as compared to the other fibroblast populations tested (Fig. 8I to 8L).

### Age-related impact of Fp and Fr on the morphology of reconstructed skin after grafting into nude mice

**Figure 9:**
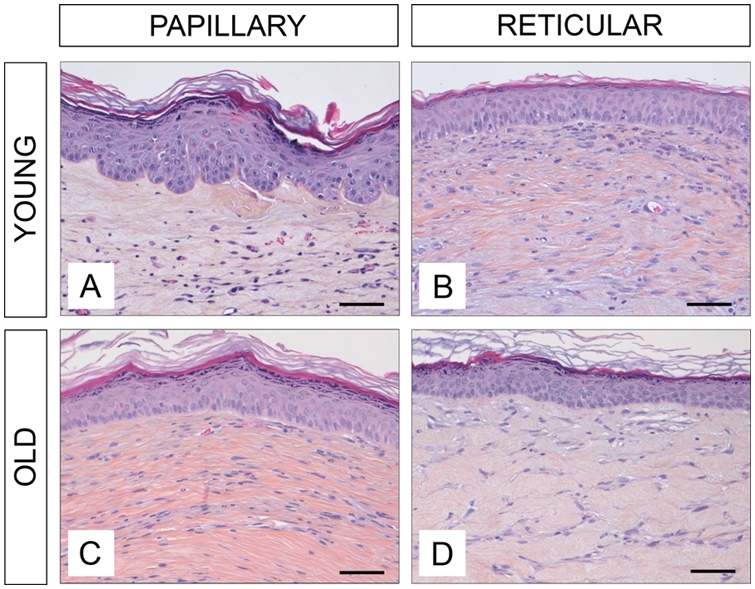
Age-dependent effects of Fp and Fr on the morphology of reconstructed skin after grafting into nude mice. The histology of the different types of three-dimensional reconstructed skin samples was next observed 5 weeks after graft onto nude mice: (A) reconstructed skin containing young Fp; (B) young Fr; (C) old Fp; (D) old Fr. All histological sections were stained with hematoxylin, eosin, and saffron (HES). Scale bars = 50 µm.

To complete the observations performed *in vitro*, similar reconstructed skin samples were grafted onto nude mice. Grafting confirmed that young Fp were more potent to promote the development of a correctly stratified and differentiated epidermis (Fig. 9A) than Fr of a similar age (Fig. 9B). In addition, we detected that under these *in vivo* conditions only, young Fp promoted the formation of rete-ridge structures in the grafted reconstructed skin samples. Rete-ridge structures were not observed in grafted skin samples containing old Fp (Fig. 9C) or old Fr (Fig. 9D). It had to be mentioned that dermal fibroblasts of recipient mice did not migrate into the dermis of human reconstructed skin after grafting during the time of experiments, as demonstrated by species specific staining of vimentin (not shown).

## Discussion

Well known characteristics of skin aging in humans are a reduction of thickness [7] and alterations in its biomechanical properties [41]. The dermis is for long thought to play a key role in this multi-causal process, since molecular studies have confirmed profound changes in the synthesis and degradation of the dermal matrix molecules with increasing age [7], [8]. Because fibroblasts which reside in the dermis can both produce and degrade extracellular matrix components, exploration of the possible evolution of their properties throughout aging is particularly important to progress in the understanding of this complex process. This study was conducted to explore the possibility that fibroblast sub-populations which reside within the superficial and the deep regions of the dermis, respectively papillary and reticular fibroblasts (Fp and Fr), may play different roles in skin aging.

We found that Fp exhibit a higher growth rate in culture than Fr, which is in agreement with previous reports [42]. However, we found that aging was associated with a reduction of this difference. Since the growth characteristics of Fr with increasing age appeared unchanged, both in term of colony growth at low culture density and in term of growth rate in mass cultures, it is the marked decrease of Fp growth potential that contributes to this leveling of the growth rates. This was taken as a first indication that Fp might be more affect by aging than Fr.

Analysis of protein secretion by the two fibroblast populations led to a similar conclusion. Most of the secreted proteins were equivalent in young and aged Fr with the exception of MCP-1, which was found down-regulated in Fr from aged donors. On the contrary, protein secretion by Fp was widely affected by aging. Whereas the growth potential of Fp decreased with age, secretion levels of the growth factor KGF, the metalloproteinases MMP-1, MMP-2, MMP-3, as well as the metalloproteinase inhibitors TIMP-1 and TIMP-2, was all increased in old compared to young Fp. Thus, the levels of different protein secretion, which were globally lower in young Fp compared to the young Fr appeared similar in Fp and Fr samples from older donors. A general increase in MMP production by dermal fibroblasts with age is described in previous reports [43]–[45]. However, none of these studies identified different secretion profiles for the specific fibroblast sub-populations, as shown here.

Our comparative studies of Fp and Fr performed at the global level of cell populations have revealed some differences in biological properties of the two fibroblast populations. However, analyses of cell morphology by flow cytometry and evaluation of colony-forming efficiency in culture have revealed that fibroblast samples from the two tissue localization present a broad heterogeneity. We routinely observed a higher proportion of fibroblasts with small size cells and low granularity in Fp samples compared to Fr samples. Dermal fibroblasts are for long known to produce clones with heterogeneous characteristics in low density cultures [23], [46]. Analysis of the clonogenic potential of Fp and Fr indicated that formation of large clones was more frequently observed in Fp compared to Fr cells. Interestingly, the high clonogenic potential of Fp faded with aged, indicating that probably only minority of the young Fp cell population accounts for the observed higher growth potential of young Fp. Loss of this particular cell subpopulation in the papillary dermal compartment during aging could be an important factor in the process of skin aging. A decrease of dermal fibroblast growth potential with age has been observed long ago [47], but the papillary localization of the fibroblasts responsible for this phenomenon was not established in this report. In this context, it is interesting to note that, in long-term cultures of fibroblasts used as an *in vitro* model of cell aging, a decrease of the growth potential [18], [19], a reduced number of small cells [48], and an increased protein production [21], have been reported, which is in agreement with our results. Fp and Fr are known to differ in their sensitivity to growth factors such as basic fibroblast growth factor (bFGF) and transforming growth factor beta 1 (TGF-β1) [37]. The observed decreased growth capacity of Fp with increasing age could result from a reduction of responsiveness to mitogenic factors, as previously suggested [24]. In a next step, comparative transcriptional profiling of Fp and Fr using full genome DNA arrays will be particularly informative to identify specific molecular characteristics of these two fibroblast populations.

The preferential evolution of the Fp population throughout aging may have important consequences on the biology of papillary dermis. In particular, reduction of the growth capacity of Fp may contribute to their decline in the papillary dermis of aged skin. Moreover, modifications of their secretion profile may have a wide impact on the biology of the tissue. Indeed, cytokines, growth factors, and MMPs/TIMPs, secreted by fibroblasts are involved in the regulation of a variety of biological processes, including inflammation [15], [49], production and remodeling of extracellular matrix components [16], [50], angiogenesis [51], as well as epithelial morphogenesis [52]. Modification of the Fp secretion profile may explain some of the structural changes in the superficial part of dermis, which have been previously associated with aging [6], [53]. For example, the simultaneous increase of MMP-1, MMP-2 and MMP-3 production in Fp may trigger the degradation of collagenous and non-collagenous components of the extracellular matrix, and consequently contribute to the deterioration of the upper dermis and basement membrane. Changes in the secretion by fibroblasts of growth factor such as KGF throughout aging may partly explain the delayed wound healing observed in elderly people [54], [55].

Recent reports have shown that dermal fibroblasts play a key role in the regulation of epidermal morphogenesis [56], [57]. In particular, these cells have been shown to influence the formation of its basement membrane [58], [59]. This prompted us to analyze the specific impact of Fp and Fr isolated from young and old adult human donors on the characteristics of three-dimensional reconstructed dermis and epidermis. Fibroblasts were embedded into type I collagen [60], and first tested for their capacity to contract lattices. We found that, whatever donor's age, Fp were less efficient in lattice contraction than Fr, which is in agreement with previous reports [61]. The capacity of Fr to contract lattices remained unchanged throughout aging unlike that of Fp, which increased with age, leading to a reduction of the functional difference between Fp and Fr in old donors. An increased capacity of fetal fibroblast to contract lattices after successive passages in culture has been reported [62], but the specific properties of Fp and Fr were not investigated in is previous study. We have secondly analyzed the effect of Fp and Fr from young and old donors on the development of an epidermis. Lattices containing Fp from young donors promoted the formation of a more correctly stratified and differentiated epidermis than Fr of a similar age. Interestingly, when these reconstructed skin samples were grafted into nude mice, only those containing young Fp formed rete ridge-like structures at the dermal-epidermal interface. It is important to note that all these particularities of young Fp were markedly decreased in Fp from old donors.

Based on these results, different hypotheses can be proposed to interpret the age-related changes of the dermal fibroblast populations in the context of skin aging. The Fp population may be progressively lost throughout aging, and be replaced by Fr. This hypothesis is comforted by previous results obtained in a reconstructed skin model, where fibroblast from the deeper dermis migrate to the upper dermis to replace superficial fibroblasts killed after UVA exposure [63]. Several lines of evidence suggest that Fr are in a more advanced state of differentiation than Fp, which leads to the alternative hypothesis that the observed decrease of Fp with age may result from a gradual transition of the Fp state to a Fr state. Thus, cellular aging may be related to a terminal differentiation process, as previously proposed [64], [65]. Such a mechanism has been described for fibroblasts derived from the upper dermis during long-term culture. Authors have shown that, after serial cultivations, these cells acquire an expression profile of collagen chains similar to that of fibroblasts isolated from the deeper part of the dermis [34].

**Figure 10:**
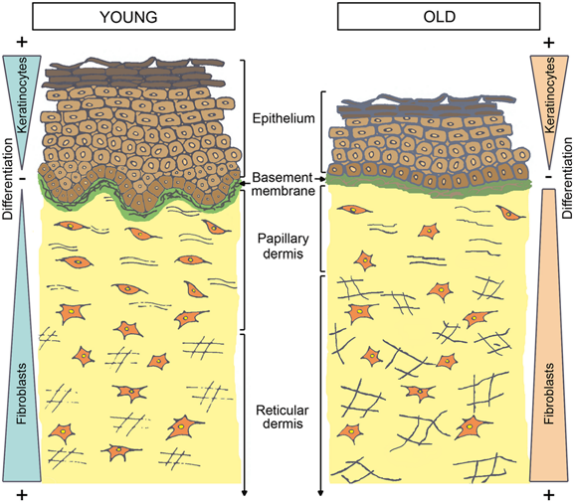
Model of skin morphogenesis and aging. Preferential modifications of Fp characteristics throughout aging may have structural and physiological consequences not only on the dermis, but also on the epidermis. Cellular differentiation of epidermal keratinocytes and dermal fibroblasts may be symmetrically organized including upward differentiation of keratinocytes associated with downward differentiation of fibroblasts. This cellular organization may be altered during skin aging.

In conclusion, our results shed a new light on the distinct properties of Fp and Fr, their modifications during aging and possible implications on skin aging. In particular, our investigations, performed at the cellular and tissue levels, revealed that the cell population defined as Fp is a central actor in this complex process. As summarized in the schema presented in Figure 10, we propose that the loss of Fp characteristics may have structural and physiological consequences not only on the dermis, but also on the epidermis. In the actual state-of-the-art, much remains to be done to understand the dynamic process leading to the specific evolution of fibroblast characteristics in particular dermal sub-localizations. The interfollicular epidermis is for long known to contain primitive subpopulations of keratinocyte with stem cell properties [66]–[70]. The more recent discovery of multipotent mesenchymal-like stem cells within the follicular [71] and non-follicular dermis [72]–[74] opens promising perspectives for future studies on dermal fibroblast origin and fate.
